# A qualitative study of contextual factors’ impact on measures to reduce surgery cancellations

**DOI:** 10.1186/1472-6963-14-215

**Published:** 2014-05-13

**Authors:** Einar Hovlid, Oddbjørn Bukve

**Affiliations:** 1 Sogn og Fjordane University College, Institute of Social Science, Postbox 133, Sogndal 6851, Norway

**Keywords:** Contextual factors, Surgery cancellations, Theoretical framework, Quality improvement

## Abstract

**Background:**

Contextual factors influence quality improvement outcomes. Understanding this influence is important when adapting and implementing interventions and translating improvements into new settings. To date, there is limited knowledge about how contextual factors influence quality improvement processes. In this study, we explore how contextual factors affected measures to reduce surgery cancellations, which are a persistent problem in healthcare. We discuss the usefulness of the theoretical framework provided by the model for understanding success in quality (MUSIQ) for this kind of research.

**Method:**

We performed a qualitative case study at Førde Hospital, Norway, where we had previously demonstrated a reduction in surgery cancellations. We interviewed 20 clinicians and performed content analysis to explore how contextual factors affected measures to reduce cancellations of planned surgeries.

**Results:**

We identified three common themes concerning how contextual factors influenced the change process: 1) identifying a need to change, 2) facilitating system-wide improvement, and 3) leader involvement and support. Input from patients helped identify a need to change and contributed to the consensus that change was necessary. Reducing cancellations required improving the clinical system. This improvement process was based on a strategy that emphasized the involvement of frontline clinicians in detecting and improving system problems. Clinicians shared information about their work by participating in improvement teams to develop a more complete understanding of the clinical system and its interdependencies. This new understanding allowed clinicians to detect system problems and design adequate interventions. Middle managers’ participation in the improvement teams and in regular work processes was important for successfully implementing and adapting interventions.

**Conclusion:**

Contextual factors interacted with one another and with the interventions to facilitate changes in the clinical system, reducing surgery cancellations. The MUSIQ framework is useful for exploring how contextual factors influence the improvement process and how they influence one another. Discussing data in relation to a theoretical framework can promote greater uniformity in reporting findings, facilitating knowledge-building across studies.

## Background

Successful quality improvement does not simply depend on the interventions implemented. The effects of interventions vary across settings, indicating that conditions besides the interventions themselves must influence the outcomes [1,2]. These conditions are referred to as contextual factors [3,4]. Øvretveit et al. have defined context as “…influences which interact with each other, and interact with the implementation process” [3, p 609]. This way of viewing context acknowledges that contextual factors and interventions influence each other. Furthermore, it recognizes that improvement often can be viewed as “facilitated evolution” [1, p i18] [5]. When researching quality improvement, it is not sufficient to demonstrate that interventions work. To facilitate the dissemination of improvements, we also need to understand why and how interventions work in particular settings, and the social mechanisms behind the interventions [6,7].

Most studies on quality improvement have been designed to evaluate the effects of interventions [3,4]. Few studies have used robust methods to assess how contextual factors influence the improvement process [3,4]. Research on contextual factors has suffered from the lack of theoretical foundations and a common framework for assessing their impact [4,8]. Hence, we have limited knowledge about how contextual factors affect improvements.

Cancellation of scheduled surgery is a quality of care problem [9,10]. We previously reported on a case where cancellations of planned surgeries were successfully reduced and improvements sustained [11,12]. The aim of this article is to contribute to our understanding of how contextual factors affect efforts to reduce surgery cancellations. We performed a qualitative analysis to explore how contextual factors influenced the improvement process in this particular case. We relate our findings to an established theoretical framework, the model for understanding success in quality (MUSIQ). Such knowledge can provide a better understanding of how surgery cancellations can be reduced. Furthermore, we discuss the usefulness of the MUSIQ framework and its possible deficits.

### Theoretical foundation

Theoretical frameworks can guide research in areas involving complex social processes that are poorly understood, including reducing cancellation of planned operations [13,14]. Such frameworks can facilitate more uniform and systematic reporting of qualitative data, enabling the development of cumulative knowledge across studies.

The importance and impact of contextual factors in quality improvement has received increasing attention [4]. Several frameworks for exploring and analyzing the effects of contextual factors on health care improvement have been developed recently. Damschroder et al. [15] developed the consolidated framework for implementation research, which consists of five domains: intervention characteristics, outer setting, inner setting, characteristics of individuals, and the process of implementation. Three domains—inner setting, outer setting, and characteristics of individuals—can be considered aspects of context [4]. Taylor et al. [16] proposed a framework that specifically addresses contextual factors related to interventions to improve patient safety. To our knowledge MUSIQ is the most comprehensive framework developed to evaluate the impact of contextual factors in quality improvement. The developers of MUSIQ state that we need conceptual models to focus and align research about the role of contextual factors [17]. Such research can provide a better understanding of how contextual factors influence improvement processes, which in turn can help practitioners manage factors important for improvement success [17,18].

In this article, we use MUSIQ as a theoretical framework for discussing the impact of contextual factors [17]. It captures most of the factors identified in the aforementioned frameworks. Furthermore, MUSIQ is not just a list of potential factors. It is built around the different organizational levels typically involved in systems improvement: organization, microsystem, and quality improvement team. Another advantage is that it addresses how contextual factors in different levels interact, and these connections have been empirically tested [19].

MUSIQ identifies 25 contextual factors that may influence the success of quality improvements. These 25 factors are structured in six main categories: external environment, organization, quality improvement support and capacity, microsystem, quality improvement team, and miscellaneous [17].

## Methods

Given that quality improvement interventions are context-sensitive, one research aim should be to explore how different configurations of contextual factors lead to different outcomes. Repeated case studies and comparisons with a framework can be used as a first step in building context-sensitive typological theories that subsequently can be tested using quantitative methods [20]. To our knowledge, the effects of contextual factors on reducing surgery cancellations have not previously been explored. Our study is therefore explorative, and we use a qualitative case study design [21]. Our case study is the redesign of the clinical pathway for elective surgery at Førde Hospital, which successfully reduced cancellations and sustained improvements to the system [11].

Førde Hospital is located in a rural community of approximately 10,000 inhabitants. The hospital offers surgery in the following disciplines: general surgery, gynecology, orthopedics, ophthalmology, otolaryngology, and odontology. It has seven operating suites and 34 surgical beds. The local health authority also includes two smaller district hospitals. Altogether, the three hospitals serve a population of approximately 107,000. Norwegian patients receive general health coverage through national state insurance, and most hospitals are publicly owned and operated.

Førde Hospital experienced high surgery cancellation rates. The management therefore initiated an improvement project to reduce cancellations spanning all surgical departments. Four improvement groups with broad clinician participation were established to recommend how to improve the clinical pathway. The main interventions included a drop-in anesthesia outpatient clinic to enable earlier and improved clinical pre-assessment of patients, a new electronic system that improved planning and patient scheduling, patient participation in surgery scheduling, and providing a telephone call to patients 2 days prior of surgery to facilitate better personalized dialog and better information [11]. These interventions were planned between September 2007 and March 2008 and implemented in March 2008. The mean cancellation rate decreased from 8.5% to 4.9% after the interventions were implemented [11]. The patients appreciated the effects of the interventions because they increased patient autonomy and participation by enabling choices that fit individual circumstances [22].

### Data collection

We interviewed employees at the hospital to explore how contextual factors influenced the improvement process. Interviews were semi-structured and based on a guide. The guide was developed through a literature review and a review of hospital administrative documents that described the aim of the improvement project and the mandate of the improvement teams. The documents were not subject to a formal document analysis. They were merely used as background information when developing the interview guide [21]. The questions in the guide covered the typical elements of an improvement project: local problem, setting, context, intended improvement, intervention planning, intervention implementation, outcomes, and efforts to sustain outcomes [23]. The guide is enclosed in the Additional file 1.

We used purposive sampling and recruited interviewees with different professional backgrounds (e.g., physicians, nurses, secretaries, and leaders) who worked in departments involved in the changed clinical pathway [24]. The interviewees’ degrees of participation in the improvement work varied. Some had participated in planning the interventions, while others were not directly engaged in the work. We invited 21 employees to participate and 20 agreed to be interviewed. We completed the interviews during June and July 2010. The interview lengths varied between 20 and 70 minutes. Seventeen interviews were conducted face-to-face, while three were conducted as telephone interviews owing to practical requirements and long travel distances in rural Norway. Each respondent was interviewed once.

### Analysis

We audiotaped the interviews, transcribed them verbatim, and transferred them to HyperRESEARCH 2.8.3 computer software (ResearchWare, Inc., Randolph, MA) for coding. We performed a content analysis using a direct approach, as described by Hsieh and Shannon [25]. EH identified and coded passages where hospital employees described how contextual factors influenced the improvement process. We derived the codes from the data [26]. OB verified the coding. Using an iterative process of coding, reflecting on the codes, and condensing, we identified common themes showing how contextual factors affected the improvement process [26]. To increase the rigor of our analysis, three key respondents validated a narrative of how interventions were planned and implemented in the hospital [27,28].

### Ethical considerations

The Western Department of the Regional Committee for Medical and Health Research Ethics in Norway deemed a full ethical review unnecessary because the study did not include experiments on humans nor did it use sensitive patient data. The study protocol was accepted by the Norwegian Social Science Data Services, which reviewed ethical aspects related to collecting and handling data, including voluntary participation based on informed consent, anonymity of informants, and presence of appropriate data storage protocols. All interviewees participated voluntarily with informed consent and could withdraw from the study at any time.

## Results

We completed interviews with 20 out of 21 employees. One interviewee withdrew consent to participate. Characteristics of the interviewees are provided in Table 1.

**Table 1:** Characteristics of the interviewees

**Professional category**	**Number of informants**	**Number participating in improvement groups**	**Number of leaders**
Physician	9	4	4
Nurse	7	5	3
Secretary	2	0	1
Administrators (project support)	2	2	1
Total	20	11	9

We identified three common themes concerning how contextual factors influenced the change process. These included 1) identifying a need to change, 2) facilitating system wide improvement, and 3) leader involvement and support. We structure the presentation of our findings around these themes and present quotes to illustrate the findings.

### Identifying a need to change

Input from patients was an important factor in identifying a need to change. Before the interventions, the hospital received complaints from patients about the pathway for elective surgery. Patients typically complained about unclear information and waiting times. Within the health care community, ideas circulate and become prevalent, for example, on the importance of patient-centered care, care pathways, and perioperative care. Prevailing ideas affected the way that local problems in this study were perceived. Leaders framed patient input in relation to these ideas. This facilitated the understanding that the pathway for elective surgery was not optimal when viewed from a patient’s perspective, and that change was necessary.

*The people behind the project believed that patients had not been given the opportunities that might be expected and that, as a consequence, the system had to be changed.* (Middle manager, in project group)

Norwegian healthcare is publicly financed, and most hospitals are owned and operated by the government, which is an important stakeholder in quality improvement in health care. Norwegian hospitals have to report to stakeholders on a number of quality indicators, including the surgery cancellation rate. The indicators from all hospitals are publicly available on the Internet. Patients can use them when they choose a provider, and they enable comparisons and benchmarking between hospitals. Førde Hospital scored below average in surgery cancellation rate before the improvement project started, which strengthened the perception that change was needed.

In the early stages of the improvement process, the organization did not have a unified understanding of what should be done. Despite these differing views, input from patients, combined with circulating ideas and national quality indicators, led to a shared understanding that change was necessary.

*Within the organization, there was a desire to try to improve the flow of patients. The disagreement was about how it was to be done, and whether it was possible.* (Middle manager, in project group)

### Facilitating system wide improvement

In 2007, the upper management of the local health care authority decided to work more systematically on general quality improvement and developed a common strategy for conducting quality improvement projects. This strategy helped to ensure a system focus throughout the improvement process.

The core aim of the strategy was to involve frontline clinicians in detecting systemic problems and subsequently to improve the corresponding clinical processes. Upper management emphasized the systemic perspective in improvement efforts by stressing that all improvement projects should address professional, patient, and management quality. The overriding objective of the improvement strategy was operationalized into understandable and manageable objectives to which clinicians could relate, including reducing surgery cancellations and ensuring the pathway to surgery was more patient-centered. As part of the strategy, a small administrative unit offering project support was established to guide clinicians in their improvement efforts.

*The main concerns have been, first, to ensure broad participation, and second, that the project should be based at the frontline level*. (Respondent from project support unit, in project group)

*When we get everybody involved together, the surgeon, the orthopedic specialist, the nurse, and the porter, and set them around the table to chart out the process and draw a process map, then something emerges. I think it has something to do with ownership.* (Middle manager, in project group)

Since no easy way to change the pathway for elective surgery was apparent, four project groups were established to suggest how various aspects could be redesigned. In accordance with the improvement strategy, a wide range of frontline professionals from different departments and with different professional backgrounds participated in the improvement groups that planned and developed the interventions. Group members were recruited partly based on their interest and willingness and partly based on their formal positions. These groups constituted a new interdisciplinary meeting place.

Group members with different professional backgrounds shared information about their daily work and related it to potential improvements in the clinical pathway for elective surgery.

*Everyone explained their workday and where they saw improvement potential.* (Respondent, in project group)

*You got to sit in a group with the doctor, the nurse, the director, and the porter, and look at all the problems. It’s not only about my challenges in dealing with a patient scheduled for an operation in an hour. There is actually an entire surrounding complex that has to function.* (Middle manager, in project group)

When group members shared information and related it to the pathway as a whole, they realized that they had worked in a fragmented way.

*The way it used to be, in many areas the big picture fell apart; work was so fragmented.* (Doctor, in project group)

*Each separate section had its own record books, and everybody tried to plan their operation schedules based on these. But there was no coordination: nothing brought things together in terms of the resources available on the ward as a whole.* (Nurse, in project group)

As professional groups in the clinical system shared information, they became increasingly aware of how different elements of the system needed to interact to improve its performance. Improvement team participants acquired a deeper understanding of the underlying causes of quality problems and why the clinical pathway had been fragmented. This new insight led to suggestions for measures to address the complexity of the problems.

*You see more than your own little task, and you see how you can become a bottleneck for other people’s tasks without even knowing it. I think that seeing the whole process, and seeing that you actually are one link in a long chain, helps people to see things more holistically.* (Respondent from project support unit, in project group)

*The most important thing I think we’ve learned is that it was very easy to sit and just look at your own sphere when working out procedures and general standards for patient scheduling. When we all sat down together and tried to create something, it required a mental readjustment so that we had to think, ‘This isn’t just about my area. It also affects others.’* (Doctor, in project group)

Things are much better now because everyone has to finish their job on the spot. Now we have procedures that require us not to release a patient with, for instance, a heart problem, on to an anaesthesiologist before they’ve been assessed by a cardiologist.

(Doctor, in project group)

Communication and information-sharing was not confined to the project groups. They communicated regularly with each other and with the relevant departments. The improvement groups had continuous dialog with clinicians outside the groups through regular meetings. All clinicians were invited to provide feedback on proposed action items in the planning phase of the interventions. Through regular meetings with health care personnel affected by the change process, leaders and project groups received feedback on the proposed actions.

*There were several information meetings along the way. People were supposed to make suggestions; they could write suggestions on pieces of paper, which were posted on the wall. They could say what they thought about the various stages in the process.* (Doctor, not in project group)

*I only see the patients when they’re with me, but I depend on their having completed the other services they need to go through before they come. So it’s easy to see the importance of a systematic plan for what the patients need to get through.* (Doctor, not in project group)

The hospital introduced a computer-based system for scheduling surgery that helped integrate surgery planning across departments and provided the overview necessary to allow patients to participate in scheduling their surgery appointment. This overview also enabled the hospital to identify problems in the clinical system that they had not been aware of previously. Information technology therefore contributed to system improvement.

*When the head of surgery looked at the clinic waiting lists, it became clear that there was an ocean of things that needed to be tackled. And these are things that we didn’t know about before because the system hadn’t been transparent.* (Middle manager, in project group)

*Now we see the big picture with regard to the operation schedule, and this means that we now discover ahead of time if two patients are scheduled for procedures that require the same instruments, and thus resterilization. This used to cause unnecessary waits.* (Nurse, in project group)

To ensure that the opportunities provided by new information technology were utilized, the upper management at the hospital created a new position: capacity coordinator. She was mandated to oversee the scheduling process across departments to ensure that total capacity was utilized in an expedient way.

The project groups received guidance and support about practical tools and improvement techniques from a small administrative unit. Such techniques helped to provide the participants with a common overview of the clinical pathway, which in turn could help identify areas for improvement.

*We drew the whole patient flow chart as it was, and then we drew a new one that illustrated what we wanted to achieve.* (Respondent, participating in project group)

### Leader involvement and support

Perceptions varied on the key actors driving the project forward. Different interviewees attributed this impetus to the top management, the middle managers, the support unit, or the clinical staff.

*Those with expertise in project and improvement measures took part throughout the preliminary investigation stage and knew exactly what had occurred before, what had been decided, and what the plans for the future were.* (Middle manager, in project group)

*You may have really good project support, but if you don’t have really good ideas, good staff, creative staff, then all you’ll get are minor adjustments or copies of what others do.* (Project support, in project group)

Interviewees agreed that the top management influenced the change process through the improvement strategy described in the previous section. They also agreed that implementing interventions was time-consuming and demanding. Middle managers had an important role in implementing and adapting the interventions. They participated in the project groups, and through their participation in the daily work process, they served as role models, monitored the degree of implementation of the interventions, and received feedback from clinicians on the need to adapt the interventions to the local context.

*I’ve learned that involving the relevant staff is not enough. Unfortunately, we need those enthusiasts* [enthusiastic middle managers] *too. This has not been a success simply because of involvement, post-it notes, and conclusions. If that were the case, we would not have progressed a single step. And that is something I think that improvement theorists need to take more seriously: that is, that the project itself is only 5 percent, or 10 percent. Ninety percent is consistent follow-up. And that is generally extremely unpleasant.* (Middle manager, in project group)

*We thought that if people had been told how to do something, they understood it. Our experience is that you almost have to be there with them and show them how we think it should be done. They need to make it their own before they can do it right.* (Nurse, in project group)

*I’ve learned that things take time. When many people are involved, you have to include them all. It’s important to talk to everyone involved and to take them seriously and try to explain why you’re doing something. Everything is connected, and we need for all the links in the chain to work.* (Respondent, in project group)

## Discussion

### Our findings

Cancellations are caused by a sub-optimally functioning clinical system [29-31]. Consequently, reducing cancellations requires changing and improving the entire clinical system. Our main finding is that different contextual factors conjointly contributed to facilitate change in the clinical system.

Change in the clinical system implies organizational change. Recently, the construct of organizational readiness for change has received increased attention. Weiner et al. [32] define organizational readiness for change as “*the extent to which organizational members are psychologically and behaviorally prepared to implement organizational change”*. Organizational readiness for change is a critical precursor to an organization’s ability to successfully implement change [32-34], and lack of organizational readiness for change is an important factor in understanding why implementation efforts fail [32,33].

One critical element for creating organizational readiness for change is establishing that the organization faces a quality problem—that there is a performance gap between the current organizational practice and a more desirable state [34-36]. Previous research has tended to frame the problem of cancellations in a management perspective, focusing on efficiency, rather than in the perspective of the patients suffering from cancellations [37-41]. In our case, input from patients was important in framing the quality problem. It created a common understanding within the organization that change was necessary. We suggest that input from patients can be an important contextual factor because it can provide a more holistic and balanced understanding of why reducing cancellations is necessary.

Reducing cancellations depends on improving the entire clinical system. A clinical system is a complex and dynamic construct, and its performance depends on how the different elements that constitute the system interact [42,43]. Previous research on reducing cancellations has not fully captured this complexity. Rather, it primarily has addressed reducing cancellations through earlier and better preoperative assessment [44-46].

We identified contextual factors that were important for facilitating system improvement in our case: interdisciplinary involvement of front line clinicians, a meeting place to enable interdisciplinary collaboration, communication with and involvement of staff not directly involved in improvement work, appropriate guidance, and expedient use of information technology.

In order to change and improve their clinical system, clinicians need to understand its interdependencies. In line with previous research, we determined that such an understanding was not present at the start of the project [47,48]. To develop this understanding, members of the clinical system need to meet and exchange information. A broad representation of different professional groups ensured that all relevant stakeholders of the clinical processes were represented in the improvement groups.

These groups represented a new meeting place and enabled a new type of interdisciplinary collaboration and information exchange. In line with the hospital’s improvement strategy, their work was framed in a systemic context, and their objective was to suggest ways to improve the clinical system as a whole. Out of this interdisciplinary collaboration emerged an improved understanding of the clinical system and its interdependencies and underlying problems. Expedient use of information technology along with guidance about improvement techniques also contributed to an overview of the clinical system and its underlying problems [49,50]. This new understanding in turn enabled the participants to plan appropriate interventions that addressed the underlying complexity of the problems.

Another factor that contributed to success was that information-sharing was not confined to improvement groups. There was extensive communication with clinicians outside the groups, which enabled external clinicians to comment on the groups’ suggestions. The new understanding of the clinical system thus spread to other parts of the organization.

In accordance with previous research, we found that involvement by upper management was an important contextual factor contributing to success [4]. Upper management facilitated system improvement through their improvement strategy. This strategy ensured that the project was anchored with upper management without compromising the professional entrepreneurship of middle managers and frontline professionals. The core aim of the strategy was to hold frontline clinicians accountable for detecting system problems and simultaneously to empower them to implement solutions. The improvement strategy provided a holistic framework for the improvement task because it required changes to address professional, patient, and management quality of care [51]. Because this overarching framework was operationalized into more comprehensible and manageable objectives such as reducing cancellations and making care more patient-centered, ownership of the change process was felt at the microsystem and team levels.

We were not able to identify a single key actor initiating and driving the improvement process. We suggest that the respondents’ inability to identify a single key actor indicates that the interdisciplinary discourse facilitated can itself be understood as a key factor driving improvement.

In our case study, middle managers were in charge of implementing the interventions devised by the project groups. The middle managers took part in the daily work processes, which allowed them to continuously monitor the degree of implementation and receive feedback from frontline clinicians on the need to re-implement and adapt interventions to the local context. Because reducing cancellations depends on changing clinical work processes, middle mangers’ participation and persistence in ensuring implementation of interventions was an important contextual factor for reducing cancellations. Dixon-Woods et al. state that having an organizational culture that is “supportive of personal and professional development” is important for success [52], [p 5]. Our findings indicate that middle managers contributed to creating a culture of supportive quality improvement, leading by example.

### Our findings in relation to the MUSIQ framework

Most of the contextual factors we identified can be related to categories of the MUSIQ framework. The different sub-categories of contextual factors in MUSIQ represent broad constructs, e.g. quality improvement leadership and maturity of organizational quality improvement. These categories need to be operationalized further and provided with empirical content, and our study can help to do so. In the following section, we discuss how the factors we identified relate to main and sub-categories of the MUSIQ framework, as outlined in Table 2.

**Table 2:** MUSIQ framework categories and contextual factors identified from our case

**MUSIQ framework categories**	**Contextual factors identified from our case**
**External environment**	• Input from patients about quality problems
• **External motivators**	• Cancellation rate as a national quality indicator
	• Circulating ideas on patient-centered care
**Organization**	• An organizational improvement strategy that provided a foundation for the improvement work by emphasizing:
• **Quality improvement (QI) leadership**	
	o Involvement of frontline clinicians
• **Maturity of organizational QI**	o Improvement by changing the clinical system
• **Culture supportive of QI**	
**Quality improvement**	• Guidance about improvement techniques and project support for the improvement teams
**support and capacity**	
• **Data infrastructure**	• Expedient use of information technology
• **Resource availability**	
**Microsystem**	• Communication with and involvement of clinicians outside the improvement teams
• **Quality improvement leadership**	
	• Middle managers’ role in following up and securing context-sensitive implementation of interventions
• **Motivation to change**	
	• Middle managers as role models participating in daily work
	• The shared belief that change was needed among hospital employees
**Quality improvement team**	• A meeting place for sharing information
• **Team diversity**	• Participation from all of the relevant professional groups in improvement teams, including physicians
• **Physicians’ involvement**	

The factors identifying a need to change correspond to MUSIQ’s main category of ‘external factors’. These factors create the consensus that change is necessary, and therefore they fall under the sub-category of external motivators. The improvement strategy and the way it was operationalized and implemented by the top management provided a foundation for improvement work on an organizational level, illustrating the importance of leadership actions at the organizational level. The strategy provided a common framework for all improvement efforts, and its focus on system improvement contributed to the maturity of the organizational improvement work.

The strategy emphasized interdisciplinary participation and was operationalized into understandable terms that clinicians at the microsystem level could relate to. These included making care more patient-centered and reducing surgery cancellations. This may have contributed to a culture supportive of quality improvement.

In line with the improvement strategy, all relevant professional groups were represented, including physicians. This team diversity, along with a structured meeting place for collaboration, was a precondition for developing a better understanding of the clinical system’s interdependencies, which was essential for planning appropriate interventions addressing the underlying problems. Expedient use of information technology and guidance about improvement techniques were also important preconditions for improving clinicians’ understanding of the clinical system. These factors can be related to data infrastructure and resource availability, which are sub-categories of quality improvement support and capacity. The teams had extensive communication with clinicians on the microsystem level who were not directly involved in the improvement teams, and received feedback on proposed actions. This communication contributed to acceptance of change.

The work in quality improvement teams was a precondition for inducing change on the microsystem level, and leadership on the microsystem level was necessary when implementing the interventions that were planned on the team level. An important finding is that we should not simply consider microsystem level factors as preconditions. Reducing cancellations of planned surgeries depends on changing the microsystem itself. The microsystem as a whole can therefore be understood as a contextual frame that must be changed in order to induce improvement. Facilitating the spread of strategies for reducing surgery cancellations to new settings requires improving our understanding of how contextual factors and interventions interact to produce change in the microsystem.

The MUSIQ framework can help us understand how factors reciprocally influence each other, because it presupposes that contextual factors at different levels affect each other, e.g. that leadership at the organizational level will influence contextual factors at the quality improvement and microsystem levels. This hypothesis is supported by our findings. Leadership by upper management is crucial for quality improvement success, but little is known about the specific actions that leaders should take and how these actions affect lower organizational levels, such as the microsystem and team levels [53]. We found that the strategies developed by upper management affected the microsystem and team levels by emphasizing the broad involvement of frontline clinicians and by providing a necessary meeting place to share information. By relating system improvement to Øvretveit’s [51] three perspectives of patient, professional, and management quality, the strategy provided a holistic framework for the kinds of information that needed to be shared and a direction for the change process. Our findings indicate that upper management actions influenced lower organizational levels by outlining a broad and commonly-accepted strategy and by facilitating the improvement team’s work, rather than by providing specific and detailed guidance for the improvement process. In other words, our study suggests that both the existence of a specified set of contextual factors, and the mechanisms by which these contextual factors reciprocally affect each other, are relevant for quality improvement success [54].

### Limitations and strengths

Our findings are based on a single case study, and should therefore be interpreted with caution. An observational and retrospective study design like ours has limitations of information bias and confounding. Our case study was exploratory. We used qualitative data, and we cannot demonstrate causality between the contextual factors and the outcomes. Moreover, we cannot specify the relative impact of the contextual factors we identified.

Damschroder et al. [15] suggest that networks and communication are important contextual factors. We found that providing meeting places to encourage sharing of information and open communication about the suggested measures were important factors contributing to acceptance and a positive climate for implementation. In our understanding of the MUSIQ framework, network and communication are factors that are not currently well covered, and we suggest that this area be developed further.

The distinction between interventions and contextual factors is not always clear-cut [1,8]. An illustrative example from our case is the way information technology was introduced and utilized. The computer application introduced for surgery planning can be regarded as an intervention. The optimal use of information technology is a recognized contextual factor that influences the success of improvement projects [55]. In our case, the way the computer application was introduced and utilized was important for the success of the project. The effect of the intervention depended on how it was introduced and adapted to the organizational context. Furthermore, the intervention itself was utilized in different ways because of how it was influenced by contextual factors. The intervention and the contextual factors thus conjointly influenced one another to promote what Øvretveit refers to as “facilitated evolution” [1]. The dynamics of how interventions and contextual factors might interact is not presently covered well by the MUSIQ framework. We suggest that more research on this topic is needed to create better models to enhance our understanding.

The strength of our study is that we investigate a case where improvements were previously demonstrated using robust statistical methods [11], and that we discuss our findings in relation to an established theoretical framework. Using a qualitative approach enabled us to explore how contextual factors affected the improvement process. We therefore supplement the MUSIQ framework with empirical data and contribute to the understanding of how it can be used. Relating data to a framework encourages uniform reporting of findings, which in turn can facilitate more cumulative and systematic knowledge-building about the impact of contextual factors across studies.

## Conclusion

Surgery cancellations are caused by the suboptimal performance of the clinical system. Consequently, reducing cancellations relies on changing the clinical system. Findings from our case study show how contextual factors interacted to facilitate change. The upper management’s improvement strategy provided a framework for system change at the microsystem level by promoting the involvement of all relevant professional groups. A meeting place where frontline clinicians could share information and develop a better understanding of the clinical system and its interdependencies enabled them to detect system problems and design adequate interventions. These findings might have relevance for hospitals trying to reduce cancellations, and might facilitate the spread of appropriate interventions by helping other hospitals to adapt these to their context. The MUSIQ framework was a useful analytic lens for exploring how contextual factors influenced the improvement process and for providing a deeper understanding of how contextual factors interacted to facilitate change. Discussing data in relation to a theoretical framework can promote greater uniformity in reporting findings, which in turn can facilitate knowledge-building across different studies.

## Competing interests

The authors declare that they have no competing interests.

## Authors’ contributions

EH designed the study, collected the data, analyzed the data, and drafted the manuscript. OB participated in the design of the study and the analysis of data, and helped to draft the manuscript. Both authors read and approved the final manuscript.

## Pre-publication history

The pre-publication history for this paper can be accessed here:

http://www.biomedcentral.com/1472-6963/14/215/prepub

## Supplementary Material

Additional file 1 Interview guide – contextual factors’ impact on measures to reduce surgery cancellations. Click here for file
