# Supplementary material for: A qualitative study of contextual factors’ impact on measures to reduce surgery cancellations
Source: BMC Health Serv Res. 2014 May 13;14:215. doi: 10.1186/1472-6963-14-215 (PMC4023492; doi:10.1186/1472-6963-14-215)
Supplement: Additional file 1 — Interview guide – contextual factors’ impact on measures to reduce surgery cancellations. [file 1472-6963-14-215-S1.docx]

# Interview guide – contextual factors’ impact on measures to reduce surgery cancellations

**Background information about interviewees:**

Gender, profession, number of years of work experience, current position, involvement in improvement work

**Quality problems**

Description of quality problems

Reasons for the quality problems

Background for current project

Prior experiences with measures to address the quality problems

Organizations’ perception of the quality problems

**Planning phase**

Ethical considerations

Reason for initiating the project

Project initiators (persons)

Improvement strategy

External factors’ impact on project planning

Organizational setting and its impact on project planning

Project organization and its relation to main organization (participants, project management, project groups)

Role of upper management

Role of middle managers

Planning and design of interventions

Background for choice of interventions

Guidance and improvement tools

Data collection

Usage of data in planning the project

Implementation of interventions (time frame, responsibility)

Adaptation of interventions

Theoretical considerations about improvement methods

Theoretical background for interventions

Involvement of organization in the planning phase

**Resources**

Usage of extra resources

Guidance

Extra positions

Spare time to work in the project

**Results**

Expected effects of interventions

Evaluation of degree of implementation of interventions

Data to evaluate effects of interventions

Mechanisms for improvement

Structural changes

Changes in work processes

Organizational setting and its impact on results (organizational change, leadership, structural elements, strategy)

Information about achieved results to main organization

Measures to sustain improvements

**Miscellaneous**

Main learning points in project

Spread and adaptation of interventions to other organizations
